# Immediate Effects of Ankle–Foot Orthosis Using Wire on Static Balance of Patients with Stroke with Foot Drop: A Cross-Over Study

**DOI:** 10.3390/healthcare8020116

**Published:** 2020-04-28

**Authors:** Jung-Hoon Lee, Im-Rak Choi, Hyun-Su Choi

**Affiliations:** 1 Department of Physical Therapy, College of Nursing, Healthcare Sciences and Human Ecology, Dong-Eui University, Busan 47340, Korea; 2 Integrated Physical Medicine Institute, Dong-Eui University, Busan 47340, Korea; 3 Department of Rehabilitation Therapy Team, Sports Exercise Therapy Center, Good Samsun Hospital, Busan 47007, Korea; irchoi@hanmail.net; 4 Department of Biomedical Health Science, Graduate School, Dong-Eui University, Busan 47340, Korea; nicecleanday@hanmail.net

**Keywords:** stroke, foot drop, static balance, ankle-foot orthosis, wire

## Abstract

The aim of this study was to investigate the immediate static balance effects of bare foot, UD-Flex ankle–foot orthosis (AFO), and AFO using wire (AOW) of patients with stroke with foot drop. Seventeen patients with stroke with foot drop (8 men and 9 women) were randomized to three conditions (bare foot, UD-Flex AFO, or AOW made with a flexible material). Static balance was assessed using the Zebris (Zebris GmbH, Isny, Germany) and BioRescue (RM Ingenierie, Rodez, France) pressure platform by a single examiner, who did not design the AOW. The order of testing with the equipment was random. The center of pressure path length (mm) measured using Zebris showed significant differences among the three conditions (bare foot, 484.47 ± 208.42; UD-Flex AFO, 414.59 ± 144.43; AOW, 318.29 ± 157.60) (*p* < 0.05). The bare-foot condition was not significantly different from the UD-Flex AFO condition (*p* > 0.05), but was significantly different from the AOW condition (*p* < 0.05). The surface area ellipse (mm^2^) measured using BioRescue showed significant differences among the three conditions (bare foot, 241.35 ± 153.76; UD-Flex AFO, 277.41 ± 381.83; AOW, 68.06 ± 48.98) (*p* < 0.05). The bare-foot condition was not significantly different from the UD-Flex AFO condition (*p* > 0.05), but the AOW condition was significantly different from the bare-foot (*p* < 0.05) and from the UD-Flex AFO conditions (*p* < 0.05). We suggest using the AOW made of flexible materials and wire instead of the UD-Flex AFO to improve immediate static balance of patients with stroke with foot drop after stroke. Further studies on the effects of dynamic balance and gait are required.

## 1. Introduction

Stroke impairs motor and sensory functions, thus causing difficulties in postural control [1], leading to postural instability and difficulties in balance and gait [2]. Functional impairment of the lower extremity affects balance and ambulation [3], and when balance is disturbed, movement is reduced, thus limiting the activities of daily living [4]. In addition to spasticity [5] and muscle weakness [6], foot drop due to plantarflexion stiffness and dorsiflexion weakness is also a major cause of poor balance in patients with stroke [7]. Foot drop occurs in 20% of stroke patients [8] and results from a weakening of the dorsiflexors or spasticity of the plantarflexors, causing reduced gait velocity, inefficient gait, and increased risk of falling [9]. Abnormal postural alignment in patients with stroke further increases asymmetry between the left and right sides of the body, thereby affecting balance, stability, and functional disability and resulting in decreased function [10]. Patients with stroke have difficulty controlling posture due to motor and sensory function abnormalities, thereby affecting balance and walking [1,2].

Ankle–foot orthosis (AFO) is the most widely used method to prevent foot drop in patients with stroke [11,12], and is used during weight-bearing training of the limb on the affected side [13] or when there is ankle spasticity or deformity [14]. It was also reported to improve abnormal gait caused by mediolateral instability of the ankle in patients with stroke [15,16] and enhance balance in patients with stroke [17,18,19]. A previous study reported that performing Biodex balance exercise for six weeks while wearing a UD-Flex AFO led to improvements in gait velocity and balance in stroke patients with foot drop compared with the use of posterior AFO [20]. UD-Flex AFO, a type of anterior AFO, is easier to wear and remove than posterior AFO; due to the open area of the calcaneus, the patient can feel direct contact of their foot with the floor when walking [20].

However, AFO passively fixes the ankle to completely restrict ankle joint movement, thus limiting mobility of the ankle joint [21,22], and was also reported to cause contracture of the ankle joint [17] and reduced muscle activity of the lower extremity [23]. It also limits ankle range of motion (ROM), which makes standing from a seated posture difficult [24]; thus, the neuromuscular system cannot be stimulated [22]. However, despite these shortcomings, plastic AFO is widely used for foot drop in patients with stroke. 

Thus, the aim of this study was to investigate the immediate static balance effects of bare foot, plastic UD-Flex AFO, and a newly developed AFO using wire (AOW) in stroke patients with foot drop.

## 2. Material and Methods

### 2.1. Patients

A sample size of 15 stroke patients with foot drop, at a significance level of 0.05, power of 80%, and effect size of 0.9 were analyzed using G-Power version 3.1 (University of Dusseldorf, Dusseldorf, Germany) [25]. The study was conducted on 17 patients, including dropouts. The selection criteria were as follows: age of 18 years or older, diagnosis of hemiplegia due to stroke, a Modified Ashworth Scale (MAS) score of ≤1 for spasticity of the lower extremity, no problems with communication, and no history of orthopedic surgery on the lower extremity. All patients gave their informed consent for inclusion before they participated in the study. The study was conducted in accordance with the Declaration of Helsinki and approved by the institutional review board at Dong Eui University (DIRB-201810-HR-E-40).

### 2.2. Study Design

**Figure 1:**
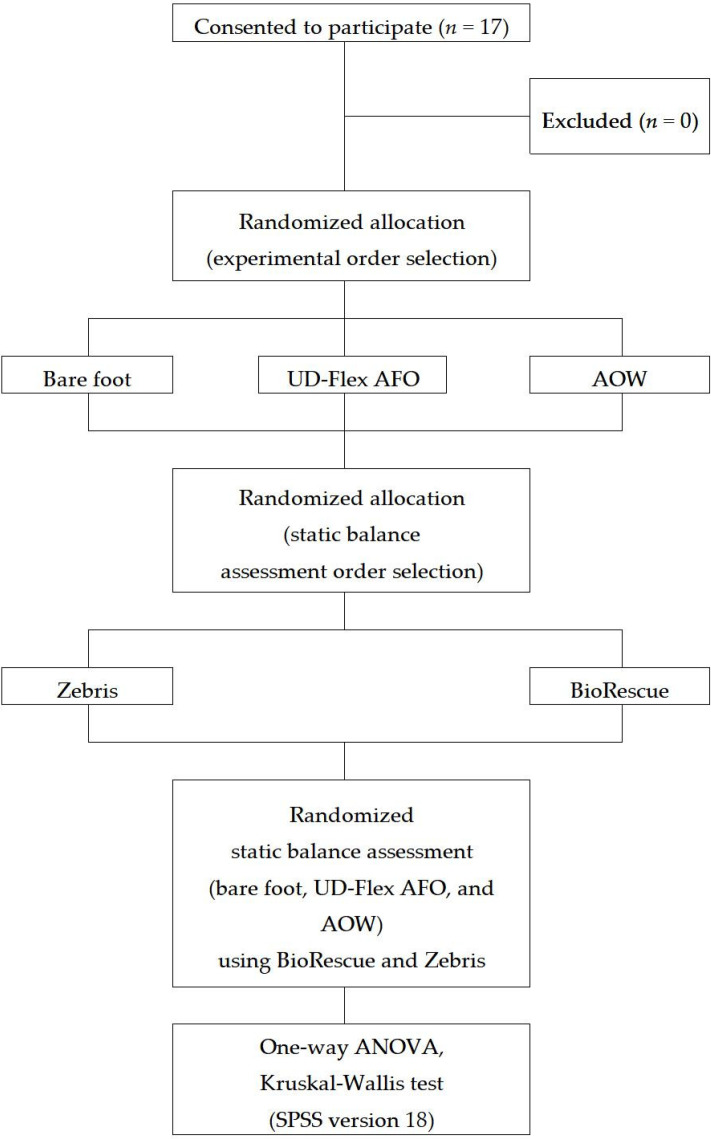
Study flowchart. AFO: ankle–foot orthosis; AOW: ankle–foot orthosis using wire; ANOVA: analysis of variance. SPSS (IBM Corp., Armonk, NY, USA).

This study used a cross-over design, with participants randomized to the bare-foot, UD-Flex AFO, or AOW conditions. All measurements were performed by the same examiner, who did not design the AOW. The examiner was blinded to the bare-foot, UD-Flex AFO, or AOW conditions in a separate space blocked by nontransparent partitions, and the data were collected via a computer connected to the measurement device. Assessment of static balance using Zebris and BioRescue was also performed by the same examiner. All participants performed static balance assessments under the three conditions. The testing order of the equipment was randomly conducted based on the order of measurement written on papers from sealed envelopes. The study flowchart of the methods and design is shown in Figure 1. 

### 2.3. Measurement

Zebris PDM-SX (Zebris GmbH, Isny, Germany) was used to electronically record and analyze the static balance and foot pressure. It consisted of a 55 cm × 40 cm × 2.1 cm (length × weight × height) platform containing 1920 activity sensors. The center of pressure (COP) path length (mm) was defined as the overall length of COP path movement during the test period [26]. In a previous study, the sway distance in the COP deceased as the posture maintenance and balance ability improved [27]. The reliability (intraclass correlation coefficient) of this equipment was 0.77–0.9 [28].

BioRescue (RM Ingenierie, Rodez, France) was used to measure the balance by computing the area of displacement (mm^2^) of the COP on a foot plate (610 mm × 580 mm × 10 mm) equipped with 1600 sensors. The lower surface area ellipse indicated better static balance [29,30]. The reliability of this equipment (intraclass correlation coefficient) ranged from 0.83 to 0.95 [31]. The static balance was measured by the participant standing with their feet shoulder-width an parallel for 30 s on the measurement plate placed 1 m away from the computer while looking straight ahead. Every time the experimental conditions were changed, participants took a 5 min rest and then lightly walked for 5 min under their respective conditions (bare foot, UD-Flex AFO, or AOW) to minimize the effects of the previous experimental condition and to adjust to the current condition.

### 2.4. Orthosis

**Figure 2:**
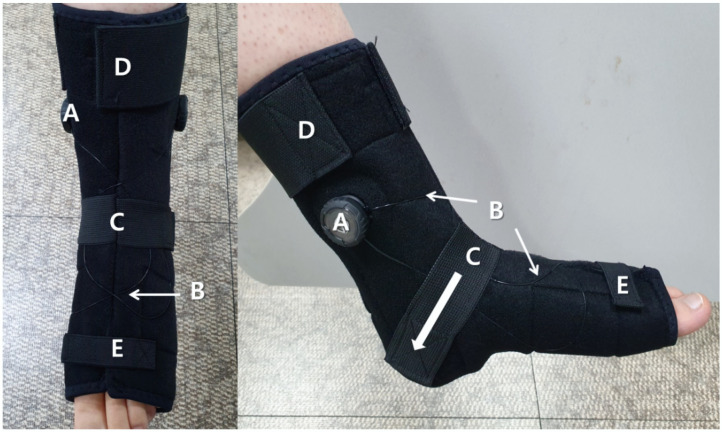
Lateral view of the ankle–foot orthosis using wire. A: wire adjustor; B: wire; C: talus posterior gliding Velcro; D: Velcro for ankle fixation; E: metatarsal joint stabilization strap.

**Figure 3:**
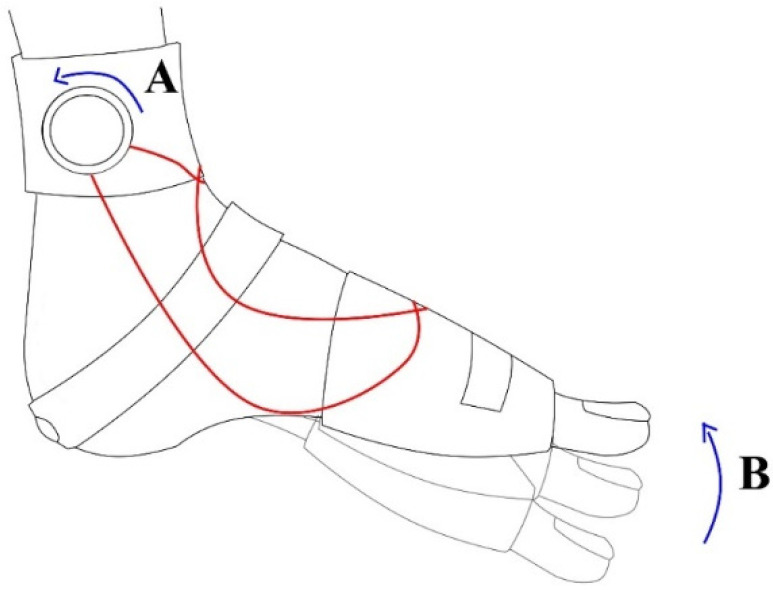
Lateral view of induction of passive ankle dorsiflexion. A: wire adjustor; B: passive ankle dorsiflexion.

The AOW (Okmeditech Co., Ltd, Changwon, Korea) is a newly developed AFO designed by the first author. It is made of a flexible material consisting of neoprene and spandex and has a polyvinyl chloride (PVC) wire to induce passive ankle dorsiflexion (Figure 2). The device was designed such that turning the wire adjustor above the lateral and medial malleoli (A in Figure 2 and Figure 3) triggers the wire crossed above the dorsum of the foot (B in Figure 2), inducing passive ankle dorsiflexion and preventing plantarflexion (B in Figure 3). Further, to assist ankle dorsiflexion, a talus strap made of polyester with rubber is stretched and attached from the front of the ankle joint toward the inferior posterior direction on both sides (C in Figure 2) to induce talus posterior gliding (Arrow of Figure 2). A Velcro strap on the top of the ankle secures the AOW from sliding down the ankle (D in Figure 2). Another Velcro strap is used to fix the front part of the orthosis above the intermetatarsal joints (E in Figure 2). 

**Figure 4:**
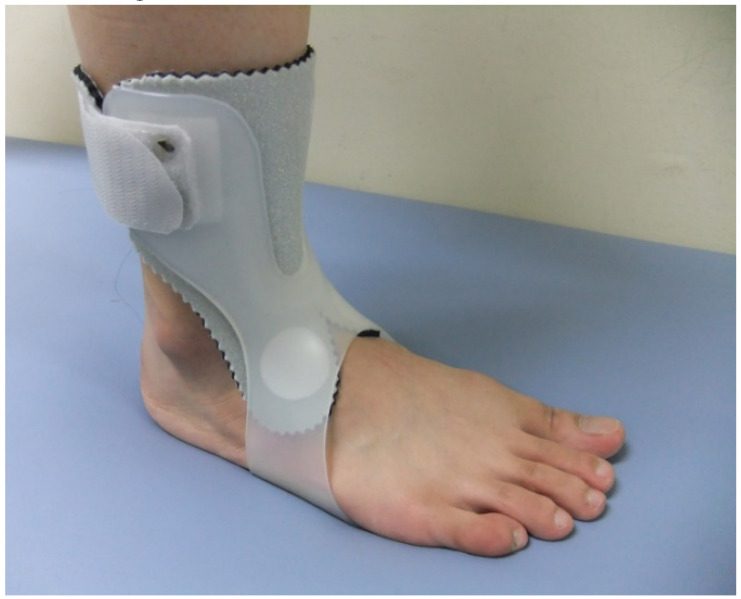
Lateral view of the UD-Flex ankle–foot orthosis.

The UD-Flex AFO used in this study was a type of plastic AFO that compensates for the shortcomings of the posterior AFOs, which is widely used in hospitals to prevent foot drop in patients with stroke (Figure 4). UD-Flex AFO is worn on the anterior part of the foot and leaves the heel open, enabling patients to feel with their heels during ambulation. Moreover, it is lightweight and small; thus, patients can easily wear and take off their shoes even while wearing the UD-Flex AFO [20]. Patients were tested while wearing AOW and AFO on their bare feet.

### 2.5. Statistical Analysis

The participants’ general characteristics were analyzed using descriptive statistics and presented as the mean and standard deviation. The data measured using Zebris were normally distributed at *p* > 0.05 as a result of normality testing using the Kolmogorov–Smirnov and Shapiro–Wilk tests. The effects of bare foot, UD-Flex AFO, and AOW on static balance were compared using one-way analysis of variance, followed by the Bonferroni test as the post-hoc test for multiple comparisons. 

Because the data obtained from BioRescue were not normally distributed according to *p* < 0.05 in the Kolmogorov–Smirnov and Shapiro–Wilk normality tests, the effects of the bare-foot, UD-Flex AFO, and AOW conditions on static balance were examined using the Kruskal–Wallis test, followed by the Mann–Whitney test as the post-hoc test for multiple comparisons. Data were statistically processed using SPSS for Windows version 18.0 (IBM Corp., Armonk, NY, USA), with the statistical significance level set at 0.05.

## 3. Results

### 3.1. Participants’ General Characteristics

Seventeen patients were enrolled in the study, and their general characteristics are shown in Table 1.

### 3.2. Static Balance Using Zebris

**Figure 5:**
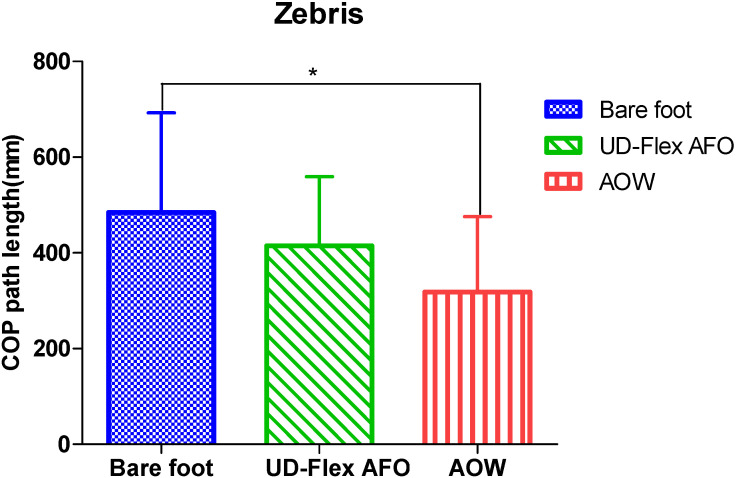
Comparisons of static balance among the three conditions using Zebris. AFO: ankle–foot orthosis; AOW: ankle–foot orthosis with wire; COP: center of pressure. * *p* < 0.05.

The COP path length measured using Zebris showed significant differences in static balance among the three conditions (*p* < 0.05) (Table 2). In the post-hoc test for multiple comparisons, no significant differences were observed between the bare-foot and UD-Flex AFO conditions (*p* = 0.470) or between the UD-Flex AFO and AOW conditions (*p* = 0.244), but significant differences were observed between the bare-foot and AOW conditions (*p* = 0.019) (Figure 5). 

### 3.3. Static Balance Using BioRescue

**Figure 6:**
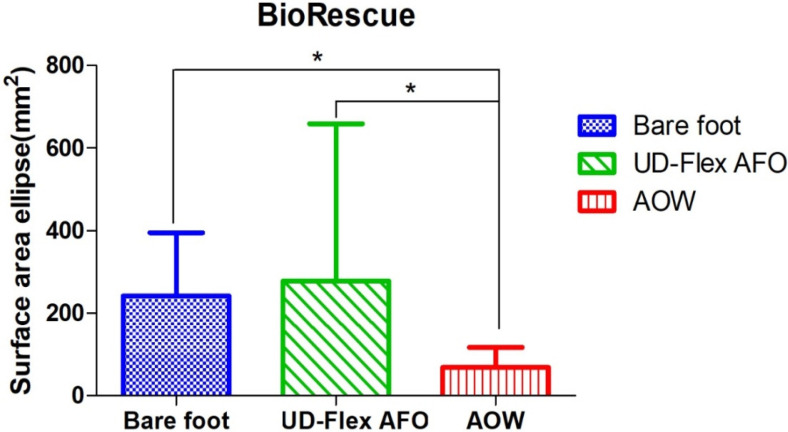
Comparison of static balance among the three conditions using BioRescue. AFO: ankle–foot orthosis; AOW: ankle–foot orthosis with wire. * *p* < 0.05.

The surface area ellipse measured using BioRescue showed significant differences among the three conditions (*p* < 0.05) (Table 3). In the post-hoc test for multiple comparisons, there were no significant differences between the bare-foot and UD-Flex AFO conditions (*p* = 0.352), but there were significant differences between the bare-foot and AOW conditions (*p* = 0.001) and between the UD-Flex AFO and AOW conditions (*p* = 0.001) (Figure 6). 

## 4. Discussion

The static balance of the UD-Flex AFO condition was not significantly increased in the BioRescue and Zebris measurements compared to the bare-foot condition. The BioRescue measurements showed significantly improved static balance when using AOW compared with the bare-foot and UD-Flex AFO conditions and the Zebris measurements showed significantly increased static balance when using the AOW compared with the bare-foot condition. AFO made with plastic limits the ROM of the ankle and decreases its mediolateral control, therefore, AFO aggravates mobility and balance [32]. Although the study of Kim et al. [33] did not focus specifically on the use of AOW, their use of an elastic band-type AFO led to improved balance compared to when a plastic AFO or bare foot was used [33]. The reason for this was suggested to be the ability of the elastic band-type AFO to promote even weight distribution between the affected and nonaffected limbs [33]. The AOW used in this study seemed to improve static balance, with the polyvinyl chloride (PVC) wire attached to the mediolateral side of the ankle potentially inducing passive ankle dorsiflexion and preventing plantarflexion, as well as promoting even distribution of pressure while decreasing COP displacement. Furthermore, the flexible material consisting of neoprene and spandex permitted minimal movement required to control the position of the ankle joint, possibly helping to improve static balance. 

Patients with stroke have trouble controlling their ankles due to the weakening of tibialis anterior, spasticity of gastrocnemius [34], and asymmetry of the anterior talofibular ligament [35], with further difficulty regarding posterior gliding of the talus below the tibia during dorsiflexion [36,37]. The lack of posterior gliding of the talus limits ankle dorsiflexion [38], which alters the alignment of the foot in turn, thereby leading to abnormal ankle movement and increased risk of ankle injury [39]. In a previous study, patients with chronic stroke who wore a flexible AFO made of elastic bands demonstrated increased balance due to the elastic band providing lesser limitation of dorsiflexion than the plastic AFO [40]. Lee et al. [41] reported that talus posterior gliding in a weight-bearing posture improved static balance in patients with stroke by increasing afferent stimulation of the ankle joint. Talus posterior gliding stimulates the afferent pathway of the mechanical receptors around the ankle joint, which enhances talocrural articulation and afferent information in the surrounding tissues [41]. Applying taping in the inferior posterior direction for talus posterior gliding increased ankle dorsiflexion in patients with limited dorsiflexion [42] and improved static balance in patients with chronic stroke [43]. The talus-stabilizing strap attached to the dorsal part of the AOW developed in this study probably functioned similarly to the taping used in previous studies [42,43], as inferior posterior gliding of the talus in a weight-bearing posture may assist ankle dorsiflexion.

This study exhibited a few limitations. First, we could not examine the effects on dynamic balance or gait, thus, we only measured static balance. Second, we did not examine the long-term effects of AOW and we only assessed immediate static balance. Third, we could not quantitatively measure the degree to which foot drop was prevented by using AOW. Fourth, we could not examine whether AOW improved static balance in stroke patients with severe spasticity. Fifth, we could not radiographically examine the function of the talus strap regarding the effects of talus posterior gliding. Sixth, only one measure of static balance was used, which made proving the validity of AOW difficult. Additional studies are needed to resolve these limitations. In a previous study on the effect of wearing an AFO on standing balance according to time since stroke occurrence, the standing balance of the group who suffered from stroke less than six months prior increased significantly, but there was no significant difference in the group who experienced stroke more than 12 months prior [17]. However, this study was not focused on the difference in the effect of the AOW on the time since the stroke occurred, which is an area in which further studies are required.

## 5. Conclusions

Our results showed that the use of AOW led to immediate effects on static balance in patients with stroke compared to those with bare feet. The use of UD-Flex AFO did not show any immediate effects on static balance in comparison with the bare-foot condition. However, further studies on the effects of dynamic balance and gait should be conducted to clinically suggest the use of AOW for stroke patients with foot drop.

## Figures and Tables

**Table 1:** General characteristics of patients (*n* = 17).

Variables	Mean ± SD or Mode (%)
Sex	
Male	8 (47.1)
Female	9 (52.9)
Age (years)	53.94 ± 14.65
Height (cm)	166.18 ± 9.47
Weight (kg)	64.76 ± 10.61
Diagnosis	
Infarction	11 (64.7)
Hemorrhage	4 (23.5)
Tumor	2 (11.8)
Affected side	
Right	10 (58.8)
Left	7 (41.2)
Modified Ashworth Scale	1.29 (0.47)
The duration of stroke (month)	12.29 (7.03)
Foot size (mm)	253.82 (12.81)
Orthosis	
UD-Flex AFO	16 (94.1)
Elastic band	1 (5.9)

**Table 2:** Static balance using Zebris.

Variables	Bare Foot	UD-Flex AFO	AOW	*p*-Value
COP path length (mm)	484.47 ± 208.42	414.59 ± 144.43	318.29 ± 157.60	0.025

Values are presented as mean ± standard deviation. AFO: ankle–foot orthosis; AOW: ankle–foot orthosis with wire; COP: center of pressure.

**Table 3:** Static balance using BioRescue.

Variables	Bare Foot	UD-Flex AFO	AOW	*p*-Value
Surface area ellipse (mm^2^)	241.35 ± 153.76	277.41 ± 381.83	68.06 ± 48.98	0.001

Values are presented as mean ± standard deviation. AFO: ankle–foot orthosis; AOW: ankle–foot orthosis with wire.
